# Waterpipe Tobacco Use in the United Kingdom: A Cross-Sectional Study among University Students and Stop Smoking Practitioners

**DOI:** 10.1371/journal.pone.0146799

**Published:** 2016-01-08

**Authors:** Mohammed Jawad, Elham Choaie, Leonie Brose, Omara Dogar, Aimee Grant, Elizabeth Jenkinson, Andy McEwen, Christopher Millett, Lion Shahab

**Affiliations:** 1 Department of Primary Care and Public Health, Imperial College London, Hammersmith, W6 8RP, United Kingdom; 2 Academic Unit of Primary Care and Population Sciences, University of Southampton, Hampshire, SO16 6YD, United Kingdom; 3 Department of Epidemiology and Public Health, University College London, London, WC1E 6BT, United Kingdom; 4 Addictions Department, Institute of Psychiatry, Psychology and Neuroscience, King’s College London, London, SE5 8BB, United Kingdom; 5 Department of Health Sciences, University of York, York, YO10 5DD, United Kingdom; 6 Institute of Primary Care and Public Health, Cardiff University School of Medicine, Cardiff, CF14 4YS, United Kingdom; 7 Centre for Appearance Research, University of the West of England, Bristol, BS16 1QY, United Kingdom; Geisel School of Medicine at Dartmouth College, UNITED STATES

## Abstract

**Introduction:**

Despite cigarette-like adverse health outcomes associated with waterpipe tobacco smoking and increase in its use among youth, it is a much underexplored research area. We aimed to measure the prevalence and patterns of waterpipe tobacco use and evaluate tobacco control policy with respect to waterpipe tobacco, in several universities across the UK. We also aimed to measure stop smoking practitioners’ encounter of waterpipe tobacco smoking.

**Methods:**

We distributed an online survey to six UK universities, asking detailed questions on waterpipe tobacco. Multivariable logistic regression models, adjusted for age, gender, ethnicity, graduate status, university and socioeconomic status (SES) assessed associations between waterpipe tobacco smoking (single use and dual use with cigarettes) and sociodemographic variables. SES was ascertained by average weekly self-spend on non-essentials. We also descriptively analysed data from a 2012 survey of stop smoking practitioners to assess the proportion of clients that used waterpipe regularly.

**Results:**

f 2217 student responses, 66.0% (95% CI 63.9–68.0%) had tried waterpipe tobacco smoking; 14.3% (95% CI 12.8–15.8%) reported past-30 day use, and 8.7% (95% CI 7.6–9.9%) reported at least monthly users. Past-30 day waterpipe-only use was associated with being younger (AOR 0.95, 95% CI 0.91–0.99), male (AOR 1.44, 95% CI 1.08–1.94), higher SES (AOR 1.16, 95% CI 1.06–1.28) and belonging to non-white ethnicities (vs. white, AOR 2.24, 95% CI 1.66–3.04). Compared to less than monthly users, monthly users were significantly more likely to have urges to smoke waterpipe (28.1% vs. 3.1%, p<0.001) report difficulty in quitting (15.5% vs. 0.8%, p<0.001), report feeling guilty, and annoyed when criticised about waterpipe smoking (19.2% vs. 9.2%, p<0.001). Nearly a third (32.5%) of respondents who had tried waterpipe had violated the UK smokefree law and a quarter (24.5%) reporting seeing health warnings on waterpipe tobacco packaging or apparatuses. Of 1,282 smoking cessation practitioners, a quarter (23.4%, 95% CI 21.5–26.1%) reported having some clients who regularly use waterpipes, but 69.5% (95% CI 67.0–72.0%) never ask clients about waterpipe use. Three quarters (74.8%, 95% CI 72.4–77.1%) want more information about waterpipe tobacco smoking.

**Conclusions:**

While two thirds of university students have ever tried waterpipe tobacco, at least monthly use is less common. Regular users display features of waterpipe tobacco dependence, and a substantial minority of SSS practitioners encounter clients who regularly use waterpipe. The lack of training on waterpipe for SSS practitioners and reported violations of smokefree laws for waterpipe highlight the need for regular surveillance of and a coordinated tobacco control strategy for waterpipe use.

## Introduction

Waterpipe tobacco smoking is a generic term to describe the inhalation of tobacco smoke after it passes through water. In the United Kingdom it is known as *shisha*, although in different settings it may be known *inter alia* as *hookah*, *narghile*, *calyan*, or *hubble-bubble*.[1] Waterpipes are currently and commonly used with a flavoured tobacco mixture heavily laced with sweeteners, honey and molasses.[2] About ten grams of tobacco is placed in the head of the apparatus, and once consumed it can be replaced with a new “head of tobacco” to enable the waterpipe session to continue. As with any tobacco smoking, waterpipe smoking exposes users to clinically harmful levels of tobacco-specific nitrosamines,[3–5] polycyclic aromatic hydrocarbons and other common toxicants found in tobacco.[6–8] Emerging evidence also suggests that users are at increased risk of cardiovascular diseases,[9, 10] lung cancer,[11] and other respiratory conditions[12, 13] relative to non-users.

Waterpipe tobacco smoking is notably prevalent in several settings.[14] Data from the most recent Global Youth Tobacco Survey (aged 13–15 years) identified high prevalence of past-30 day waterpipe use in Lebanon (36.9%), the West Bank (32.7%) and Latvia (22.7%).[15] The National Youth Tobacco Survey (aged 11–18) in the US suggests past-30 day waterpipe prevalence has grown from 4.1% to 9.4% between 2011–2014.[16] Among adults in Europe, prevalence of regular or occasional use are highest in Latvia (11.5%), Lithuania (9.0%), Cyprus (8.5%) and Denmark (8.4%).[17] Only three UK studies have measured waterpipe prevalence in adults. Among university students, research showed that between 38–52% had ever tried waterpipes and between 8–11% were past-30 day users,[18, 19] and among a general population sample in Great Britain 28% of the 18–24 age group had ever tried waterpipes and 3.5% were frequent users.[20] Such UK studies offer limited further insight in the epidemiology of waterpipe use due to the lack of detailed waterpipe behavioural measures.

High waterpipe tobacco prevalence can be explained by several factors. Users often perceive this flavoured product to be relaxing, entertaining, attractive and socially acceptable, resulting in reduced harm perception.[21, 22] Other influences include its availability and affordability,[21] misleading industry marketing campaigns,[23] and the lack of evidence-based interventions to promote cessation.[24]

Despite the popularity of waterpipe tobacco among youth worldwide, detailed data are lacking from countries such as the UK owing to its omission from routine national health surveys. There is a need to understand how waterpipe tobacco fits with the changing tobacco epidemiology among young people, such as whether risk factors are similar for waterpipe-only smokers and dual users who smoke cigarettes as well as waterpipes, and whether patterns of waterpipe consumption behaviour differ between regular and non-regular users. There is also little evidence on the effectiveness of legislation on waterpipe tobacco smokers, which is important considering flavoured waterpipe tobacco will be exempted from the soon-to-be-implemented European Tobacco Products Directive which will ban flavoured cigarettes.[25] One review of the national statutes from 62 countries worldwide highlighted possible exemptions from health warning label requirements on waterpipe tobacco,[26] and in countries where these exemptions do not exist, waterpipe tobacco companies remain non-compliant.[27] Only a few published randomised controlled trials exist of waterpipe cessation interventions, which show promise in favour of behavioural support,[28, 29] however it is unknown whether smoking cessation services are routinely encountering clients who smoke waterpipe tobacco and who wish to cease use.

Given these research gaps, this study aimed to measure the prevalence of waterpipe tobacco smoking among university students in the UK, and compare correlates between waterpipe-only and dual waterpipe/cigarette users. This study also aimed to explore the waterpipe patterns of behaviour, including frequency of use and measures of dependence. We wanted to explore whether waterpipe smokers had ever violated the English smokefree law (comprehensive smoking ban inside ‘substantially enclosed’ public places), recalled noticing health warnings, and experienced misleading advertising from waterpipe-serving premises. Finally, this study aimed to explore the extent to which waterpipe tobacco users engage with stop smoking practitioners.

## Methods

This study was approved by the IRB/ethics committees of Imperial College London, University College London, King's College London, University of York, University of the West of England and Cardiff University

### Design, Sample, Setting

We conducted a cross-sectional study of six convenience-sampled universities across the United Kingdom. Three universities were situated in London, a city known to have a high number of waterpipe-serving premises (approximately 400),[30] and a higher than average prevalence of use.[20] The remaining three universities were from other large UK cities (Cardiff, Bristol, York) with unknown waterpipe tobacco smoking prevalence. Our sample frame was comprised of enrolled undergraduate and postgraduate students at these universities.

Between 2013 and 2014 one researcher from each university sought ethical approval and distributed an online, self-administered survey. Recruitment methods were not identical between universities due to logistical practicalities: most (n = 4) used one university-wide listserv, one used a departmental listserv, and one posted a notice to the university-wide electronic bulletin board lasting three weeks, which students could choose to view as part of the university’s online working environment. The initial recruitment email (or message, in the case of bulletin board notices) contained a short message explaining the purpose of the study and a link to the online survey. The landing page of the online survey provided further details on the rationale and objectives of the study. It was made clear that starting the survey constituted informed consent, and that participant could email the lead researcher should they wish to withdraw from the survey and have their data deleted. Participants had to provide their university email addresses to verify their student status and to allow identification of duplicate entries. Email addresses were deleted from the dataset prior to analysis to maintain anonymity.

We also conducted a secondary analysis of the 2012 Annual Survey of Stop Smoking Practitioners. This online, self-administered, 60-item survey was distributed by email to all stop smoking practitioners registered between 2010 and 2012 with an online training programme (www.ncsct.co.uk) and contracted to work in the NHS Stop Smoking Service. It was also distributed to those who had completed a similar survey in the previous year and not registered for training, and distributed to all managers of the 152 English NHS stop smoking services. Further details of this survey can be found elsewhere.[31]

### Measures

**Fig 1:**
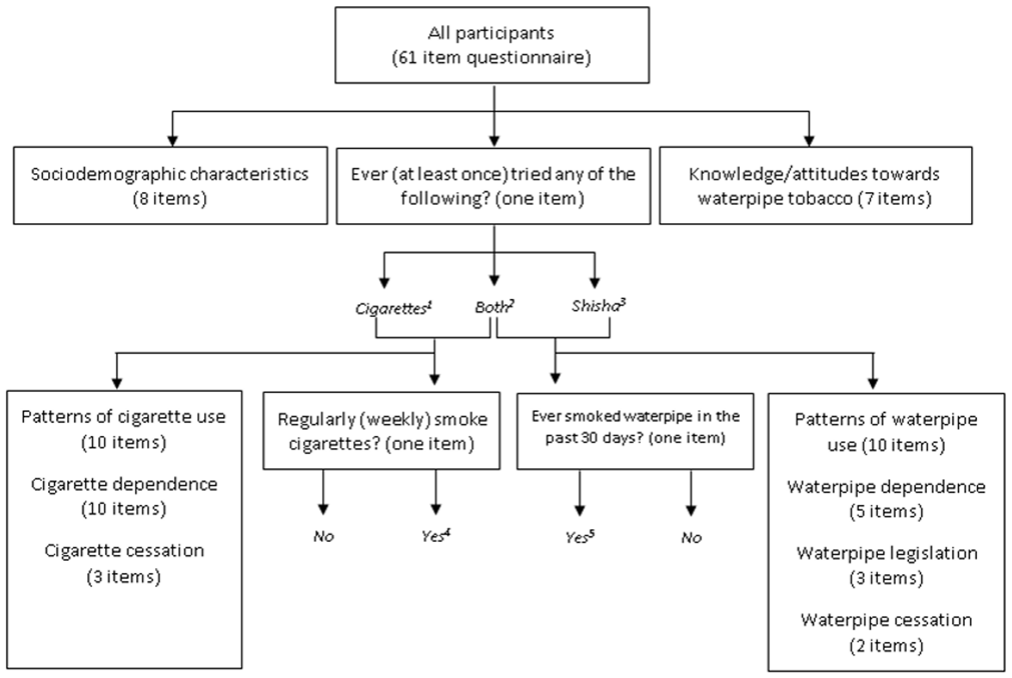
Questionnaire structure and outcome measures. Legend: Outcome measures: 1+2: Ever cigarette use; 2+3: Ever waterpipe use; 2: Ever dual use; 4: Current cigarette use; 5: Current waterpipe use; 4+5: Dual current use.

For university students we distributed a 61-item questionnaire. Its structure and key outcome measures are described in Fig 1. All respondents answered questions on sociodemographic characteristics, knowledge and attitudes towards waterpipe tobacco and the following: “Have you ever (at least once) smoked any of the following, even just one or two puffs?” (Cigarettes/Shisha/Both/No). Those answering “shisha” or “both” were considered to have ever tried waterpipe tobacco, and those answering “cigarettes” or “both” were considered to have ever tried cigarettes. Those answering “Yes” to the question “Have you smoked shisha at least once in the last 30 days?” were considered past-30 day waterpipe tobacco users, and those answering “Yes” to the question “Do you regularly (weekly) smoke cigarettes?” were considered current cigarette users. Current dual use was defined as both past-30 day waterpipe use and current cigarette use. Other questions included the patterns of tobacco use, tobacco dependence (urges to smoke, difficulty in quitting), tobacco cessation (tried quitting, needing help to quit) and waterpipe legislation (violation of smoking ban, recall of health warnings, misleading health messages).

Covariates used in analysis were age, gender, ethnicity (white/non-white), and graduate status (undergraduate/postgraduate). Weekly expenditure was assessed as follows “In an average week, how much money do you spend on yourself (other than for essentials)?” (<£10/£10-20/£20-30/£30-40/£40-50/>£50) and served as a proxy for socioeconomic status. Other variables of interest included the frequency of tobacco use, dependence measures, cessation attempts, initiation location and provider, unconventional waterpipe use (mixing alcohol with water in the base of the apparatus, or drugs with the tobacco), and evaluative measures of waterpipe tobacco policy (such as smoking waterpipe inside public venues and exposure to health warning labels).

For stop smoking practitioners, we were interested in three questions included in the 60-item survey. These were “To the best of your knowledge, out of 100 clients that you see how many use the waterpipe regularly? (please indicate number between 0 and 100)”, “Would you like more support and information about waterpipe use?” (Yes or No), and “What proportion of your clients do you ask whether they smoke waterpipe?” (None of them, Very few of them, Some of them, Most of them, or All of them).

### Statistical analysis

Waterpipe prevalence outcome measures were calculated as a proportion of the total sample. We cross-tabulated each outcome measure by covariates to calculate prevalence of waterpipe tobacco use by sociodemographic characteristics. We tested the association between each outcome measure and covariates by logistic regression, adjusting for the university variable using fixed effects models (State command: logistic). Data from the survey of stop smoking practitioners were described descriptively. We reported adjusted odds ratios and their 95% confidence interval, taking a significance level of 5%. We controlled for the family-wise error rate using the false discovery rate method[32]. All analyses were conducted in Stata 12 (StataCorp).

## Results

### Characteristics of sample

We deleted observations that were conducted by staff (n = 3) as we only aimed to recruit students. Due to the study design it was not possible to calculate a response rate. A total of 2213 student responses were analysed, and their sociodemographic characteristics are shown in Table 1. Over half of the sample derived from one university in London. As a whole, the sample was in their early twenties, mainly female and mainly belonging to white ethnic groups. Most participants were undergraduates, and about half spent between £10–30 a week on themselves. Fifteen percent considered themselves weekly cigarette users, and two thirds had ever tried cigarettes.

**Table 1:** Participant characteristics (N = 2213).

Characteristic	
	Mean (SD)
**Age**	23.4 (5.9)
	**% (N)**
**University**	
University College London	*59*.*6 (1318)*
Cardiff University	*17*.*8 (394)*
University of the West of England (Bristol)	*6*.*2 (136)*
University of York	*6*.*0 (133)*
Imperial College London	*5*.*8 (129)*
King’s College London	*4*.*7 (103)*
**Gender**	
Female	*58*.*4 (1257)*
Male	*41*.*6 (897)*
**Ethnicity**	
White	*74*.*9 (1613)*
Non-white	*25*.*1 (541)*
**Educational level**	
Undergraduate	*59*.*9 (1291)*
Postgraduate	*40*.*1 (863)*
**Weekly expenditure**	
<£10	*13*.*0 (277)*
£10–20	*27*.*3 (584)*
£20–30	*23*.*1 (493)*
£30–40	*16*.*6 (354)*
£40–50	*10*.*5 (224)*
>£50	*9*.*6 (204)*
**Current (weekly) cigarette use**	
No	*84*.*9 (1676)*
Yes	*15*.*1 (299)*
**Ever tried cigarettes**	
No	*34*.*2 (731)*
Yes	*65*.*8 (1404)*
**Past-30 day waterpipe use**	
No	*85*.*6 (1789)*
Yes	*14*.*4 (300)*
**Ever tried waterpipes**	
No	*34*.*0 (726)*
Yes	*66*.*0 (1409)*

### Prevalence and correlates of waterpipe tobacco use

A total of 66.0% (95% CI 64.0–68.0%; n = 1409) reported having ever tried waterpipe tobacco, and 14.4% (95% CI 12.9–15.9%; n = 300) reported past-30 day waterpipe tobacco use. The majority of those who had ever tried waterpipes, had tried cigarettes and waterpipes (83.4%; 95% CI 81.4–85.3%; n = 1175) and 16.6% (95% CI 14.6–18.6%; n = 234) reported having ever tried waterpipe only. The majority of those who reported past 30-day waterpipe use, reported past-30 day only use (70.7%; 95% CI 65.5–75.8; n = 212) and 29.3% (95% CI 24.2–34.5%; n = 89) reported past-30 day dual use. Of the full sample, 23.3% (95% CI 21.5–25.1%; n = 497) had never tried either waterpipe tobacco or cigarettes.

Tables 2 and 3 present the prevalence and correlates of waterpipe tobacco smoking by sociodemographic characteristics. Waterpipe tobacco smoking was significantly higher in younger age groups, males, and among those having higher weekly expenditure. Postgraduate students were more likely to have tried both waterpipe and cigarettes compared to undergraduate students. Associations between waterpipe tobacco smoking and ethnic group showed inconsistent patterns. Ever trying waterpipe tobacco only, past-30 day waterpipe-only use and current dual use was higher in non-white ethnic groups, but ever trying waterpipe and cigarettes was higher in white ethnic groups.

**Table 2:** Prevalence of waterpipe tobacco smoking by characteristics, % (n).

Characteristic	Ever tried waterpipe only^1^	Past-30 day waterpipe only use^2^	Ever tried both^3^	Current dual use^4^
**TOTAL**	***11*.*0 (234)***	***10*.*1 (212)***	***55*.*0 (1175)***	***4*.*2 (89)***
**Gender**				
Female	*10*.*8 (135)*	*8*.*7 (106)*	*52*.*4 (653)*	*3*.*2 (39)*
Male	*11*.*2 (99)*	*12*.*2 (106)*	*58*.*7 (522)*	*5*.*7 (50)*
**Ethnicity**				
White	*8*.*6 (138)*	*7*.*9 (124)*	*59*.*1 (947)*	*3*.*8 (60)*
Non-white	*18*.*0 (96)*	*16*.*8 (88)*	*42*.*8 (228)*	*5*.*5 (29)*
**Graduate status**				
Undergraduate	*10*.*3 (132)*	*10*.*7 (135)*	*53*.*2 (679)*	*5*.*4 (68)*
Postgraduate	*11*.*9 (102)*	*9*.*3 (77)*	*57*.*7 (496)*	*2*.*5 (21)*
**Weekly expenditure**				
<£10	*14*.*9 (41)*	*8*.*7 (24)*	*39*.*1 (108)*	*3*.*3 (9)*
£10–20	*10*.*5 (61)*	*7*.*8 (45)*	*49*.*0 (286)*	*2*.*8 (16)*
£20–30	*10*.*3 (51)*	*9*.*8 (47)*	*59*.*2 (292)*	*5*.*0 (24)*
£30–40	*9*.*6 (34)*	*11*.*3 (39)*	*65*.*8 (233)*	*3*.*7 (13)*
£40–50	*11*.*2 (25)*	*12*.*0 (26)*	*55*.*4 (124)*	*4*.*5 (10)*
>£50	*10*.*8 (22)*	*15*.*6 (31)*	*64*.*7 (132)*	*8*.*5 (17)*

^1^at least one or two puffs of waterpipe in lifetime and never tried cigarettes;

^2^used waterpipe at least once in the last 30 days and non-current cigarette user;

^3^at least one or two puffs of waterpipe tobacco and cigarettes;

^4^at least one waterpipe in the last 30 days and at least weekly cigarette use

**Table 3:** Correlates of waterpipe tobacco smoking and dual use with cigarettes, by characteristics.

Characteristic	Ever tried waterpipe only (n = 234)	Past-30 day waterpipe only use (n = 212)	Ever tried both (n = 1175)	Current dual use (n = 89)
	*AOR (95% CI)*, *p-value*			
**Age**^§^	*0*.*96 (0*.*93*, *1*.*00)*, *p = 0*.*09*	*0*.*95 (0*.*91*, *0*.*99)*, *p = 0*.*03*	*0*.*96 (0*.*94*, *0*.*98)*, *p<0*.*001*	*0*.*95 (0*.*89*, *1*.*01)*, *p = 0*.*15*
**Gender**				
Female	*1*.*00*	*1*.*00*	*1*.*00*	*1*.*00*
Male	*1*.*05 (0*.*79*, *1*.*39)*, *p = 0*.*78*	*1*.*44 (1*.*08*, *1*.*94)*, *p = 0*.*03*	*1*.*29 (1*.*08*, *1*.*55)*, *p = 0*.*03*	*1*.*76 (1*.*13*, *2*.*72)*, *p = 0*.*03*
**Ethnicity**				
White	*1*.*00*	*1*.*00*	*1*.*00*	*1*.*00*
Non-white	*2*.*27(1*.*70*, *3*.*03)*, *p<0*.*001*	*2*.*24 (1*.*66*, *3*.*04)*, *p<0*.*001*	*0*.*49 (0*.*40*, *0*.*60)*, *p<0*.*001*	*1*.*44 (0*.*90*, *2*.*31)*, *p = 0*.*17*
**Graduate status**				
Undergraduate	*1*.*00*	*1*.*00*	*1*.*00*	*1*.*00*
Postgraduate	*1*.*45 (1*.*01*, *2*.*09)*, *p = 0*.*07*	*1*.*19(0*.*80*, *1*.*77)*, *p = 0*.*44*	*1*.*35 (1*.*08*, *1*.*70)*, *p = 0*.*03*	*0*.*60 (0*.*32*, *1*.*13)*, *p = 0*.*15*
**Weekly expenditure**^§^	*0*.*92 (0*.*84*, *1*.*01)*, *p = 0*.*11*	*1*.*16 (1*.*06*, *1*.*28)*, *p = 0*.*03*	*1*.*24 (1*.*16*, *1*.*31)*, *p<0*.*001*	*1*.*27 (1*.*10*, *1*.*47)*, *p<0*.*03*

**Note**: for definition of prevalence measures see Table 2 footnote; model adjusted for university;

^§^left as continuous variable, where AOR is the change in the independent variable following one unit change (for age: 1 year; for weekly expenditure: £10 spend) in the dependent variable

### Patterns of use and policy evaluation

Table 4 presents descriptive results of other features of respondents who had ever tried waterpipe tobacco. Over three quarters of respondents who had ever tried waterpipe tobacco smoked less than monthly (57.2%) or once (19.5%), and only 1.0% of them smoked daily. Over half the sample reported initiating waterpipe smoking in a shisha café, and nearly three quarters were introduced to it by a friend. Regarding policy evaluation measures, a third reported ever violating the smokefree law since its implementation, a quarter ever noticed health warnings on waterpipe tobacco packaging or on waterpipe apparatuses, and one in ten reported ever being informed of the safety of waterpipe smoking from shisha café staff or marketing material.

**Table 4:** Other features of respondents who had ever tried waterpipe tobacco.

Feature	Ever tried waterpipe (N = 1406)
	% (n)
**Behaviour**	
Frequency of waterpipe use	
Don’t smoke anymore	*9*.*2 (125)*
Smoked once	*19*.*5 (266)*
Less than monthly	*57*.*2 (780)*
Monthly	*9*.*0 (123)*
Weekly	*4*.*2 (57)*
Daily	*1*.*0 (13)*
**Initiation**	
Location of first waterpipe	
Shisha café	*54*.*1 (738)*
Friend’s house	*27*.*0 (368)*
Other	*18*.*9 (258)*
Provider of first waterpipe	
Friend	*73*.*7 (996)*
Self	*12*.*3 (167)*
Relative	*8*.*0 (108)*
Other	*6*.*0 (84)*
**Policy**	
Public indoor waterpipe use in the UK since smokefree law implementation	
No	*62*.*1 (837)*
Can’t remember	*5*.*5 (74)*
Yes	*32*.*5 (438)*
Noticed health warnings on waterpipe tobacco packaging or apparatus	
No	*75*.*5 (1019)*
Yes	*24*.*5 (330)*
Safety of waterpipe communicated by café staff or marketing materials	
No	*89*.*7 (1210)*
Yes	*10*.*3 (139)*

Table 5 presents further descriptive results stratified by frequency of use (less than monthly vs. at least monthly). Most respondents reported smoking either one or two heads of tobacco per session, 15.1% reported mixing alcohol with the water and 11.3% reported mixing the tobacco with cannabis. An analysis of the 2.4% respondents who specified mixing ‘other’ substances showed that these mainly included soft drinks, energy drinks, and milk. These measures show no consistent difference between less than monthly and at least monthly users. Compared to less than monthly users, those using at least monthly were significantly more likely to have urges to smoke waterpipe (3.1% vs. 28.1%, χ^2^ p<0.001), report difficulty in quitting waterpipes (0.8% vs. 15.5%, χ^2^ p<0.001), report feeling annoyed when people criticised waterpipe smoking habits or told them to quit waterpipe (9.5% vs. 32.2%, χ^2^ p<0.001), report feeling guilty about waterpipe smoking (9.2% vs. 19.2%, χ^2^ p<0.001), and report ever having tried to stop smoking waterpipe (4.3% vs. 12.4%, χ^2^ p<0.001).

**Table 5:** Other features of respondents who had ever tried waterpipe tobacco, stratified by frequency of use.

Feature	Ever tried waterpipe (N = 1406)	Less than monthly use (N = 778)	At least monthly use (N = 193)	
	% (n)			p-value*
**Number of heads per session**				
1	*48*.*8 (666)*	*60*.*9 (474)*	*60*.*6 (117)*	*0*.*99*
2	*19*.*3 (263)*	*23*.*1 (180)*	*27*.*5 (53)*	*0*.*24*
3	*3*.*2 (44)*	*2*.*8 (22)*	*6*.*2 (12)*	*0*.*05*
4	*0*.*5 (7)*	*0*.*5 (4)*	*1*.*6 (3)*	*0*.*17*
Don’t know	*11*.*0 (150)*	*12*.*1 (94)*	*4*.*2 (8)*	*0*.*001*
N/A (smoked once only)	*17*.*2 (234)*	*0*.*5 (4)*	*0*.*0 (0)*	*0*.*37*
**Has mixed the other substances with waterpipe**				
Alcohol	*14*.*7 (207)*	*15*.*9 (124)*	*20*.*7 (40)*	*0*.*15*
Cannabis	*11*.*0 (154)*	*12*.*1 (94)*	*16*.*6 (32)*	*0*.*13*
Other	*2*.*1 (30)*	*2*.*4 (19)*	*5*.*2 (10)*	*0*.*08*
**Presence of urges in last 24 hours**				
No	*94*.*2 (1271)*	*96*.*9 (754)*	*71*.*9 (138)*	*<0*.*001*
Yes	*5*.*8 (78)*	*3*.*1 (24)*	*28*.*1 (54)*	
**Strength of urges in last 24 hours**				
Slight	*73*.*2 (52)*	*95*.*8 (23)*	*61*.*7 (29)*	*0*.*02*
Moderate to very strong	*26*.*8 (19)*	*4*.*2 (1)*	*38*.*3 (18)*	
**Felt need to cut down but found it difficult**				
No	*96*.*0 (983)*	*99*.*2 (726)*	*83*.*9 (156)*	*<0*.*001*
Yes	*4*.*0 (41)*	*0*.*8 (6)*	*16*.*1 (30)*	
**Feels annoyed when people criticise habits or tell to quit**				
No	*84*.*8 (587)*	*90*.*5 (417)*	*67*.*8 (118)*	*<0*.*001*
Yes	*15*.*2 (105)*	*9*.*5 (44)*	*32*.*2 (56)*	
**Feels guilty about waterpipe smoking**				
No	*86*.*7 (1160)*	*90*.*8 (702)*	*80*.*9 (152)*	*<0*.*001*
Yes	*13*.*3 (178)*	*9*.*2 (71)*	*19*.*2 (36)*	
**Ever tried to stop waterpipe smoking**				
No	*90*.*8 (871)*	*95*.*7 (665)*	*87*.*6 (162)*	*<0*.*001*
Yes	*9*.*2 (88)*	*4*.*3 (30)*	*12*.*4 (23)*	
**Needed help/support to stop waterpipe smoking**				
No	*95*.*4 (83)*	*100*.*0 (30)*	*95*.*7 (22)*	*0*.*29*
Yes	*4*.*6 (4)*	*0*.*0 (0)*	*4*.*4 (1)*	

*Chi-squared test for differences in proportion between less than monthly use and at least monthly use

#### Stop smoking practitioners

Of 1,282 stop smoking practitioners, a quarter (23.4%, 95% CI 21.5–26.1) reported having some clients who reported that they had regularly used waterpipes, of whom the median percentage of clients who used waterpipe regularly was 3% (IQR 1–7%, range 1–85%) per practitioner. However, 69.5% (95% CI 67.0–72.0%) of practitioners never asked clients about waterpipe use. Three quarters (74.8%, 95% CI 72.4–77.1%) of practitioners wanted more information about waterpipe tobacco smoking.

## Discussion

### Main findings

In this sample of approximately 2,000 university students, two thirds had tried waterpipe tobacco smoking and 15% reported past-30 day waterpipe use. Waterpipe use was higher among younger groups, males, and students of high socioeconomic status, and past-30 day users were more likely to belong to non-white ethnicities. The conversion rate from ever trying waterpipe to daily or weekly use is very low at around 5%, so it is unlikely to be as dependence-inducing as other substances (e.g. cigarettes). While most students who had ever tried waterpipe had also tried cigarettes, most past-30 day waterpipe smokers were non-current cigarette smokers. Most users of waterpipe tobacco smoked waterpipe tobacco intermittently. A third had ever violated the smokefree law and a quarter recalled noticing health warnings on waterpipe tobacco packaging or the waterpipe apparatus. A small but considerable proportion experimented with using alcohol or cannabis in the waterpipe apparatus.

Waterpipe tobacco smoking is perceived by adolescents and young adults as a trendy, fashionable and socially acceptable health behaviour,[21, 22] which is the likely driving force for experimentation.[14] Given the main location of use are relatively expensive waterpipe-serving premises (usually a restaurant, bar or café), this may explain the association between waterpipe tobacco use, higher socioeconomic status and intermittent patterns of use. Our policy evaluation findings are likely to be due to ongoing legislation enforcement difficulties among waterpipe-serving premises in the UK,[30] which may be the result of a lack of direct waterpipe-specific guidance in statute e.g. how to enforce health warning labels on waterpipe apparatuses.[26]

### Previous research

Studies from the US suggest between 33–48% of university students have ever tried waterpipe smoking, and 10–22% are past-30 day users.[33–36] Looking closer at frequency of use, studies of university students in the UK identified that the proportion of at least weekly use among past-30 day waterpipe users varied between 26–52%.[18, 19] and among university students in the US showed that of those who had tried waterpipe smoking, 42% were at least monthly users.[37] These proportions are much higher than our estimates and may reflect temporal patterns: a longitudinal study among female university students in the US showed that frequent waterpipe use occurs at the start of the academic year,[38] presumably a time which coincides with increased socialising.

Previous studies have documented the use of unconventional substances in waterpipes, however the extent to which this occurs is unknown. In one qualitative study among young adults in London, all regular waterpipe users either partook or heard of others engaging in this form of experimentation.[39] In the US, a survey among 3,447 college students revealed that 45% of waterpipe users used the apparatus to smoke marijuana, and 18% used it to smoke hashish.[40] Our reported level of 11% is lower than this and could be explained by underlying cultural norms towards recreational drug use. Our estimates are similar to a study among 90 waterpipe users in Saudi Arabia, where 18.9% mixed the apparatus water with soft drinks, and 7.8% added flowers, spices, or drugs to the tobacco.[41] A qualitative analysis with local governments in London highlighted that several waterpipe-serving premises openly advertise ‘alcoholic waterpipes’, usually at premium prices.[30]

### Public health implications

While waterpipe tobacco smoking appears to be a prevalent but infrequent activity, longitudinal studies indicate that it may serve as a gateway for future cigarette use among adolescents in the US[42] and Jordan.[43, 44] Until research explores this relationship further, it is important that tobacco control efforts are not undermined by the growing interest in waterpipes. It is therefore imperative that national surveillance, including the use of standardised measures of prevalence to enable comparative analyses,[45] is implemented for this product.

This study also highlights the difficulty in estimating harm exposure resulting from waterpipe tobacco smoking. Waterpipe tobacco smoking sessions are often 30–45 minutes in duration[37, 46] (sometimes up to several hours[39]) and harm exposure is likely to be a function of the number and depth of puffs.[47] Although we asked about the number of heads per session, around one in ten were unsure of how many heads they smoked. Future waterpipe prevalence surveys should consider including measures to estimate harm exposure, such as the frequency of sharing the pipe with others, the mean length of each session and the number of heads per session. Meanwhile stop smoking practitioners should be provided information about waterpipe tobacco and be urged to routinely ask about its use. Evidence for effective cessation interventions are few but show promise in favour of behavioural interventions.[28, 29]

### Policy implications

Given our findings, several waterpipe tobacco policy actions need to be addressed. While waterpipe-serving premises are included under England’s comprehensive smokefree law,[26] about a third of those who had ever used waterpipes have smoked inside such premises since its implementation. Evidence from one qualitative study among local government identified that an unintended consequence of the smokefree law was the deliberate and recurrent non-compliance of waterpipe-serving premises, compounded by the lack of resources to enforce it.[30] Of concern, air quality in these venues is considered to be poorer than for venues where cigarette smoking was once permitted indoors.[48]

Smoking waterpipe in commercial settings is unlikely to expose users to health warning labels, as the apparatus is prepared by staff and presented to the user in a pre-packaged form. There have been persistent calls for guidance in enforcing health warning labels on the waterpipe apparatus and related accessories.[26, 27, 30] Finally, consideration should be given to waterpipe-serving premises serving ‘alcoholic waterpipes’, of which there are at least three in London.[30] Only Turkey has statutory legislation banning the use of liquids other than water in the base of the waterpipe apparatus; however, the impact of this policy remains unevaluated.[26]

### Strengths and weaknesses

This is the first multi-centre study of waterpipe tobacco smoking among university students in the UK, benefitting from a large sample size and diverse set of questions that provide useful insights into its patterns of use. We did not conduct probability sampling and recruitment methods varied across universities. While this may introduce selection bias, our sampled ratio of males to females (1:1.4) and non-white to white students (1:3) is similar to the 2013/2014 enrolment data from the UK Higher Education Statistics Agency (male to female: 1:1.1; non-white to white 1:3.5).[49] We over-recruited from one London university; however, analyses were adjusted for the university of each respondent. As this is a cross-sectional analysis, we cannot make any causal claims about the direction of associations. Although data were self-reported, it is unlikely to have introduced biased responses given the anonymity of the surveys. Weekly expenditure may not accurately measure socioeconomic status given that spending and the ability to spend may not always correlate in the student population. However, studies using other proxy measures of socioeconomic status similarly identify the relationship between waterpipe use and high socioeconomic status.[14, 20, 50]

## Conclusions

A large proportion of university students have ever tried waterpipe tobacco, although most used it intermittently or once only. Current users are more likely to be younger, wealthier males from non-white ethnicities. Unconventional use of waterpipe smoking is not uncommon and warrants further attention. A substantial minority of SSS practitioners encounter clients who regularly use waterpipe. The lack of relevant SSS training and reported violations of smokefree laws highlight the need for regular surveillance of and a coordinated tobacco control strategy for waterpipe use.

Universities should incorporate health education measures in order to raise awareness of the harms associated with waterpipe use. Policy makers should respond to these findings by ensuring adequate guidance is given for the enforcement and enactment of waterpipe tobacco legislation to be placed on par with cigarettes. Further surveillance is needed to understand the extent to which existing tobacco control efforts may be undermined by the growing interest in waterpipe tobacco.

## Supporting Information

S1 File Raw dataset used for university student questionnaire. (DTA)

S2 File Raw dataset used for stop smoking practitioner questionnaire. (DTA)
